# Area-specific economic status should be regarded as a vital factor affecting the occurrence, development and outcome of cervical cancer

**DOI:** 10.1038/s41598-020-61660-5

**Published:** 2020-03-16

**Authors:** Zichao Li, Haozhi Wu, Xiaowei Yi, Fangyu Tian, Xiyang Zhang, Haikun Zhou, Biqing Liu, Zhenhua Lu, Jing Wang, Dongbo Jiang, Lei Shang, Kun Yang

**Affiliations:** 1 Department of Immunology, Air Force Medical University, Xi’an, China; 2 Department of Burns and Cutaneous Surgery, Xijing Hospital, Air Force Medical University, Xi’an, China; 3 West China School of Public Health and West China Fourth Hospital, Sichuan University, Chengdu, China; 4 Brigade of Cadet, Air Force Medical University, Xi’an, China; 5 North China University of Technology, Beijing, China; 6 Department of Health Statistics, School of Public Health, Air Force Medical University, Xi’an, China

**Keywords:** Health care, Public health

## Abstract

For patients with cervical cancer, despite the incidence and mortality rates have been declining in recent years, due to its huge population base, cervical cancer has always been a serious public health problem. Our research placed emphasis on the indices greatly associated with overall area-specific social economic status, making up for the defects of traditional research which only pay attention to the situation of some specific disease or patients’ individual social status. A total of 39160 women identified cervical cancer were concluded in our study from the Surveillance, Epidemiology, and End Results (SEER) 18 Program data between 1980 and 2014. With improving the area-specific social economic factors in recent years, the occurrence and prognosis of cervical cancer showed different variation patterns respectively. Some states like California and Georgia for their better economic status and more healthcare investment by local medical institution, population there showed a lower prevalence, incidence, more timely diagnosis, effective treatment, and better prognosis. According to our study, we aimed to give a scientific interpretation on how the area-specific social economic factors affect the disease situation at the macro level and help local medical institution make advisable decisions for controlling cervical cancer.

## Introduction

Cervical cancer is the fourth most frequent diagnosed cancer type^1^ and the third leading cause of cancer deaths among females in the world. In United States, it was also regarded as an important public health problem, for nearly 250,000 women currently live with the cervical cancer^2^, 12,820 new cases’ occurrence and 4,210 patients’ deaths annually^3^.

With the progress in research targeting to cervical cancer, infection of the Human Papilloma virus (HPV) was confirmed as a vital etiologic risk factor of cervical cancer. Due to the promotion of first screening, the incidence and the mortality rates decreased gradually though early intervention and reduction of the risk factors^4^. As a result, cervical cancer became a preventable disease in general population.

In recent years, more and more women got benefits from medical development under the support by local economic institution, for spreading HPV vaccine since 2006^4^ and applying many advanced treatment gradually. However, in some underdeveloped areas, most women still suffered undiagnosed and had limited access to proper treatment or prevention in their daily life^5–8^. These differences might derive from many aspects. According to previous studies, variation of patients’ incidence rates and outcome have been greatly associated with ages, tumor histology, tumor differentiation^7,9,10^, and individual socioeconomic status, including race/ethnicity and insurance status^11–13^. However, the overall social economic factors have been ignored, which may be greatly associated with development of local health care infrastructure construction and advanced technologies. In order to better understand vital features of cervical cancer at a population-based level and conduct area-specific cancer control programs, we have done the further exploration for related factors of happening and prognosis of cervical cancer in our longitudinal disease-relevant study. Moreover, our research would guide a direction for preventing and controlling the cervical cancer, and conducting better health interventions in different states of US. in the future.

Data information was obtained from the Surveillance, Epidemiology, and End Results (SEER) Program at the National Cancer Institute (NCI)^7^. GDP and area-specific health care expenditure were collected by State, both of which were sourced from Centers for Medicare & Medicaid services government site (CSM.gov) https://www.cms.gov/index.html, to estimate the local overall economic condition and medical construction https://www.bls.gov/.

## Results

### Prevalence analysis for the cohort

The prevalence of cervical cancer associated with patients’ demographic data and condition of tumor were presented in Supplementary Table 1. The prevalence of diseases showed a declining trend from 1980 to 2014. Meanwhile, as is demonstrated in that table, with the passage of time, the prevalence existed significant different trends in our long-term research (*P* < 0.01) among all the presented variables including different races, age groups, histological types, differentiated stages and grades, and registered states. The African-Americans between 40–59 years old were more likely to suffer from cervical cancer. Additionally, among all patients, the majority was diagnosed at stage I, grade II and III.

### Incidence disparities in time series analysis

Table 1 presented the distributions of cases’ incidence characteristics in our study. Table 1 and Supplementary Figure 1 showed the disparities of the incidence rates on cervical cancer among women during 1980–2014 related to patients’ demographic data and status of the disease in nine states. Patients diagnosed at the middle age group (40–59 years) were significantly higher than mentioned other age groups (*P* < 0.001). The African Americans had a larger declining trend from 12.3 to 4.2 per 100,000 women which was most distinctive among the groups (*P* < 0.001). In addition, significant differences in incidence also existed among different tumor status (*P* < 0.001). A majority of patients were diagnosed as stage I (1.6 per 100,000 women), grade II or III (3.0 per 100,000 women). According to the annual percent changes (APCs) showed in supplementary Table 2, patients at early stages kept down trend slopes in our time series analysis. On the contrary, patients diagnosed at stage III and stage IV increased after 1995. For the geographic factors, the incidence of the cervical cancer showed the different disparities among 9 states. The incidence rates of cervical cancer in Utah, California, Connecticut, and Washington during 1980–2014 are much lower than that in Georgia, Michigan and New Mexico. The largest declining slopes were showed in California and Georgia in the long period, and their average annual percent changes (AAPCs) for incidence rates were both −2.3%. By contrast, AAPCs for cervical cancer cases in Hawaii was only −1.4% (Supplementary Table 2).

**Table 1:** Age-standardized cervical cancer incidence rates among women ages 20+ years in 9 states during 1980–2014.

		1980–1984		1985–1989		1990–1994		1995–1999		2000–2004		2005–2009		2010–2014		1980–2014		*P*
		Count	Rate	Count	Rate	Count	Rate	Count	Rate	Count	Rate	Count	Rate	Count	Rate	Count	Rate	Rate
Race	Caucasians	4,393	5.2	4,468	5.0	4,664	4.8	4,375	4.2	3,853	3.6	3,646	3.4	3,669	3.3	29,068	4.1	<0.001
	African Americans	916	12.3	886	10.2	867	8.5	909	7.7	806	6.1	739	4.9	715	4.2	5,838	7.0	
	Others	407	6.7	519	6.9	557	5.8	672	5.7	607	4.2	576	3.4	673	3.3	4,011	4.6	
Ages	20–39	1,716	5.0	1,887	5.0	1,946	4.8	1,844	4.5	1,486	3.8	1,382	3.7	1,343	3.5	11,604	4.3	<0.001
	40–59	2,002	9.0	2,073	8.8	2,295	8.3	2,539	7.8	2,366	6.4	2,265	5.7	2,398	6.0	15,938	7.1	
	60+	2,002	12.4	1,938	10.9	1,875	9.9	1,624	8.2	1,444	6.9	1,353	5.8	1,382	5.0	11,618	8.0	
Histology	Squamous cell carcinoma	2,699	2.8	2,590	2.5	2,471	2.1	2,436	1.9	2,297	1.7	2,340	1.6	2,411	1.6	17,244	2.0	<0.001
	Adenocarcinoma	480	0.5	650	0.6	771	0.7	797	0.6	726	0.5	716	0.5	827	0.6	4,967	0.6	
	Others	1,718	1.7	1,795	1.7	1,912	1.6	1,747	1.4	1,299	1.0	969	0.7	903	0.6	10,343	1.2	
Surgery	Performed	2,765	3.9	3,301	4.2	3,962	4.6	4,100	4.4	3,347	3.4	2,925	2.9	2,857	2.7	23,257	3.7	<0.001
	Not recommended	2,426	3.6	2,304	3.2	2,079	2.6	1,815	2.1	1,897	2	1,988	1.9	2,185	2	14,694	2.4	
Stage	Stages I			1,375	1.8	3,355	3.9	3,428	3.7	2,266	2.3	2,419	2.4	2,371	2.3	10,424	1.6	<0.001
	Stages II			372	0.5	801	1	776	0.9	606	0.6	651	0.6	607	0.5	2,555	0.4	
	Stages III			317	0.4	873	1.1	807	0.9	701	0.7	884	0.9	1,004	0.9	2,698	0.4	
	Stages IV			198	0.3	535	0.7	498	0.6	484	0.5	591	0.6	776	0.7	1,715	0.3	
Grade	Grade I	402	0.6	392	0.5	414	0.5	508	0.6	463	0.5	455	0.5	549	0.5	3,183	0.5	<0.001
	Grade II	1056.00	1.50	1189.00	1.60	1357.00	1.60	1470.00	1.60	1465.00	1.50	1,474	1.5	1,603	1.5	9,614	1.5	
	Grade III	1,074	1.60	1,317	1.80	1,504	1.80	1,504	1.70	1,409	1.50	1,384	1.4	1,375	1.3	9,567	1.5	
	Grade IV	129.00	0.20	106.00	0.10	163.00	0.20	140.00	0.20	134.00	0.10	122	0.1	107	0.1	901	0.1	
State	California	887	5.6	948	5.5	976	5.2	935	4.6	737	3.4	672	3.0	767	3.2	5,922	4.2	<0.001
	Connecticut	792	5.2	808	5.1	792	4.7	802	4.6	655	3.6	594	3.2	653	3.5	5,096	4.2	
	Georgia	534	7.3	550	6.3	599	5.8	701	5.8	616	4.5	607	4.1	613	3.6	4,220	5.0	
	Michigan	1,252	7.2	1,171	6.5	1,140	5.9	1,114	5.6	962	4.7	861	4.3	745	3.7	7,245	5.3	
	Hawaii	211	5.1	261	5.5	297	5.3	304	5.1	265	4.2	265	4.0	261	3.7	1,864	4.5	
	Iowa	796	6.0	720	5.4	725	5.2	676	4.7	573	3.9	527	3.5	538	3.6	4,555	4.5	
	New mexico	382	6.8	396	6.1	444	6.1	400	4.8	415	4.6	386	4.0	389	3.8	2,812	4.9	
	Washington	650	5.0	775	5.2	806	4.6	782	3.9	778	3.7	803	3.5	848	3.5	5,442	4.1	
	Utah	234	4.2	281	4.4	353	4.8	314	3.6	305	3.2	298	2.7	298	2.7	2,101	3.5	

Source: SEER-NLMS Record Linkage Study. Based on the registered population among 9 SEER Stares (California, Connecticut, Georgia, Hawaii, Iowa, Michigan, New Mexico, Utah and Washington) during 1980–2014.

^a^Rates were per 100000 and age-adjusted to the 2000 US standard population by the direct method.

^b^Rates were estimated by Join point regression models.

^c^Wilcoxon rank-sum tests were used to identify the different variation of incidence among different races, age groups, histology types, surgery performed status and states over time; Linear-by-Linear association tests were used to identify the different variation of incidence among tumor differentiated stages and grades over time.

**Figure 1:**
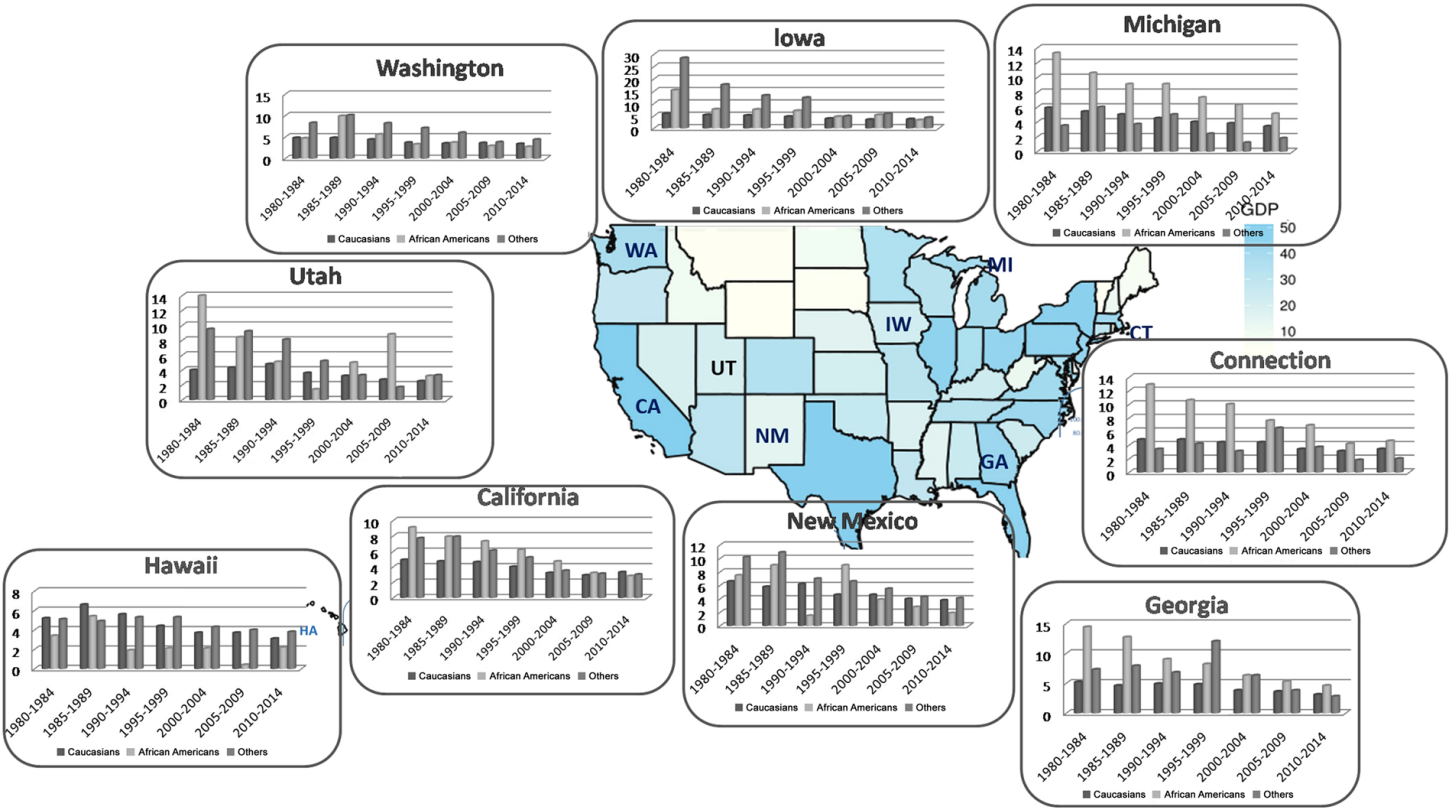
The differences of variation tendency for age-adjusted  incidence rates among patients registered in 9 states were showed in histogram. The geographic heat map was colored according to the GDP rank status in the recent years.

**Figure 2:**
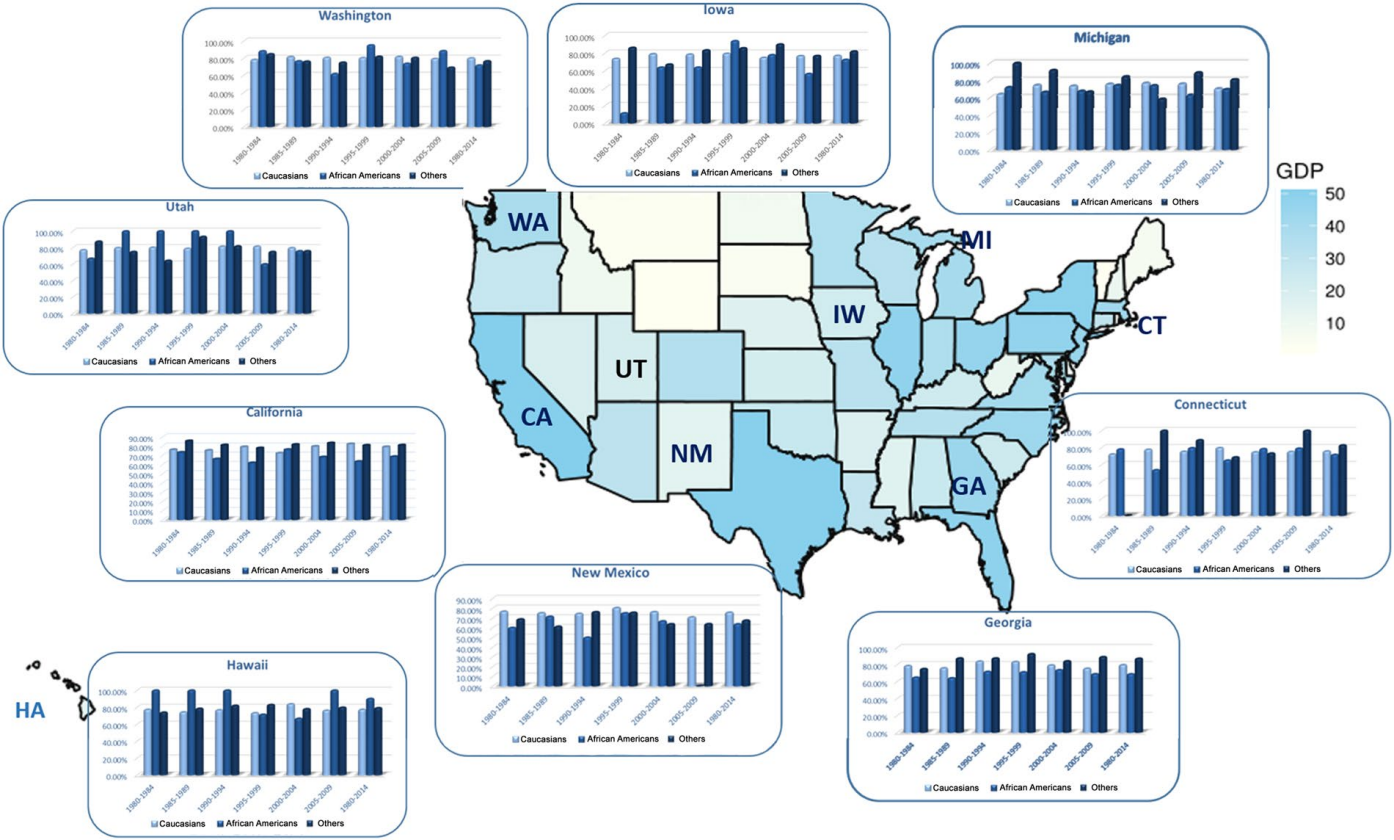
The histogram showed trends of age-adjusted 3-year cause specific survival rates disparity ratios by registered region and race/ethnicity between 1980 and 2014 for registered population. The geographic heat map was colored according to the GDP rank status in the recent years.

Through analyzing patients’ demographic data on races among 9 states, significant differences in variation of incidence rates existed over time in Fig. 1. For each economic or health care expenditure imparity, the variation status for categorized covariates parts in 9 areas differed from one to another (Fig. 2). Between 1980–2014, area-specific GDP, PHC expenditure, Medicare expenditure and Medicaid expenditure were all closely related to cervical cancer incidence rates among nine registry states, whose Pearson correlation coefficients were using |*r* | ranging from 0.6994 to 0.9531(*P* < 0.001). With the GDP index and health care expenditure increasing, the incidence rates of the cervical cancer cases had significant downward slopes. Strong relation existed between variation tendency of incidence rates and social economic factors in the following seven registry states: California, Connecticut, Georgia, Iowa, Michigan, New Mexico, and Washington, whose correlation coefficients were greater than 0.8 (*P* < 0.001). In Hawaii and Utah, the association between these factors and incidence trends were moderate relevant whose correlation coefficients were between 0.6–0.8 (*P* < 0.001), which were all showed in Supplementary Figure 2.

### Prognosis and outcome of patients with cervical cancer

**Figure 3:**
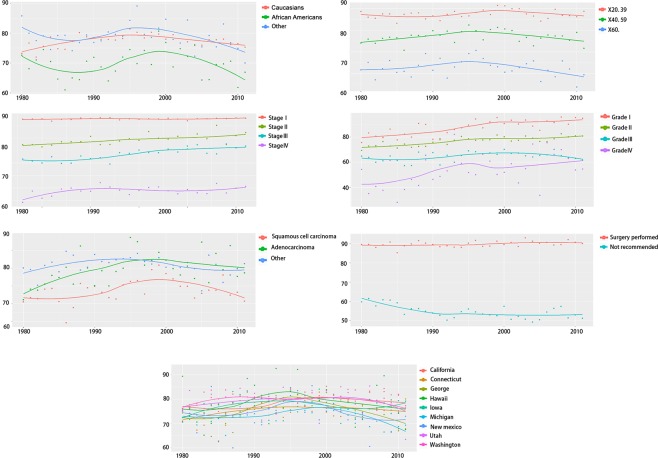
The variation tendency of age-adjusted 3-year CSS rates of registered patients with cervical cancer during 1980–2014 according to following variables: race/ethnicity, ages at diagnosis, tumor stage, tumor grade, tumor histology, surgery performed status and registered states.

Through analyzing the 3-year CSS rates on the basis of different variables and different periods with Friedman test, all these variables listed in the Supplementary Table 3 were statistically significant (*P* < 0.001), which means that the influence on patients’ prognosis for respectively demographic and pathological factors changed greatly over time. Long-term disparities on 3-year CSS rates were depicted through using LOESS curves in Fig. 3. Distinctive upward trends of 3-year CSS rates were showed and women with highly differentiated tumor always gained the most benefit in the long period. In addition, despite factors such as diagnosed ages, different races, tumor histology, surgery status and registered states for 3-year CSS rates fluctuated around the baseline during the 31 years, the significant differences of variation between these factors still existed (*P* < 0.001) (Fig. 3 and Supplementary Table 3). For our collected patients, 3-year CSS rates had a slight improving in early years, and latterly, the slope presented a down-trend. Due to patients’ different races among 9 states, significant differences existed for the variation of 3-year CSS rates, which were showed in Fig. 2.

Local GDP index, PHC expenditure, Medicare and Medicaid expenditure showed the poor correlation with 3-year CSS rates of cervical cancer cases using Pearson correlation method at the state level (|*r* | < 0.4, *P* > 0.05), and the details showed in Supplementary Figure 3. The correlation between GDP index and 3-year CSS rates of patients in California, Connecticut, Georgia, Hawaii, Iowa and GDP index presented the positive correlation, while the negative correlation existed in Michigan, New Mexico, Utah and Washington. In California, Connecticut, Hawaii and Iowa, with the increasing of the expenditure for the personal health care and Medicare, the 3-year CSS rates showed up-trend slopes throughout the long period as well; on the other hand, long term down-trend slopes were presented in the other 5 states. Additionally, the Medicaid showed the positive correlations among California, Connecticut and Georgia, and negative correlations were showed for Hawaii, Iowa, Michigan, New Mexico, Utah and Washington.

**Figure 4:**
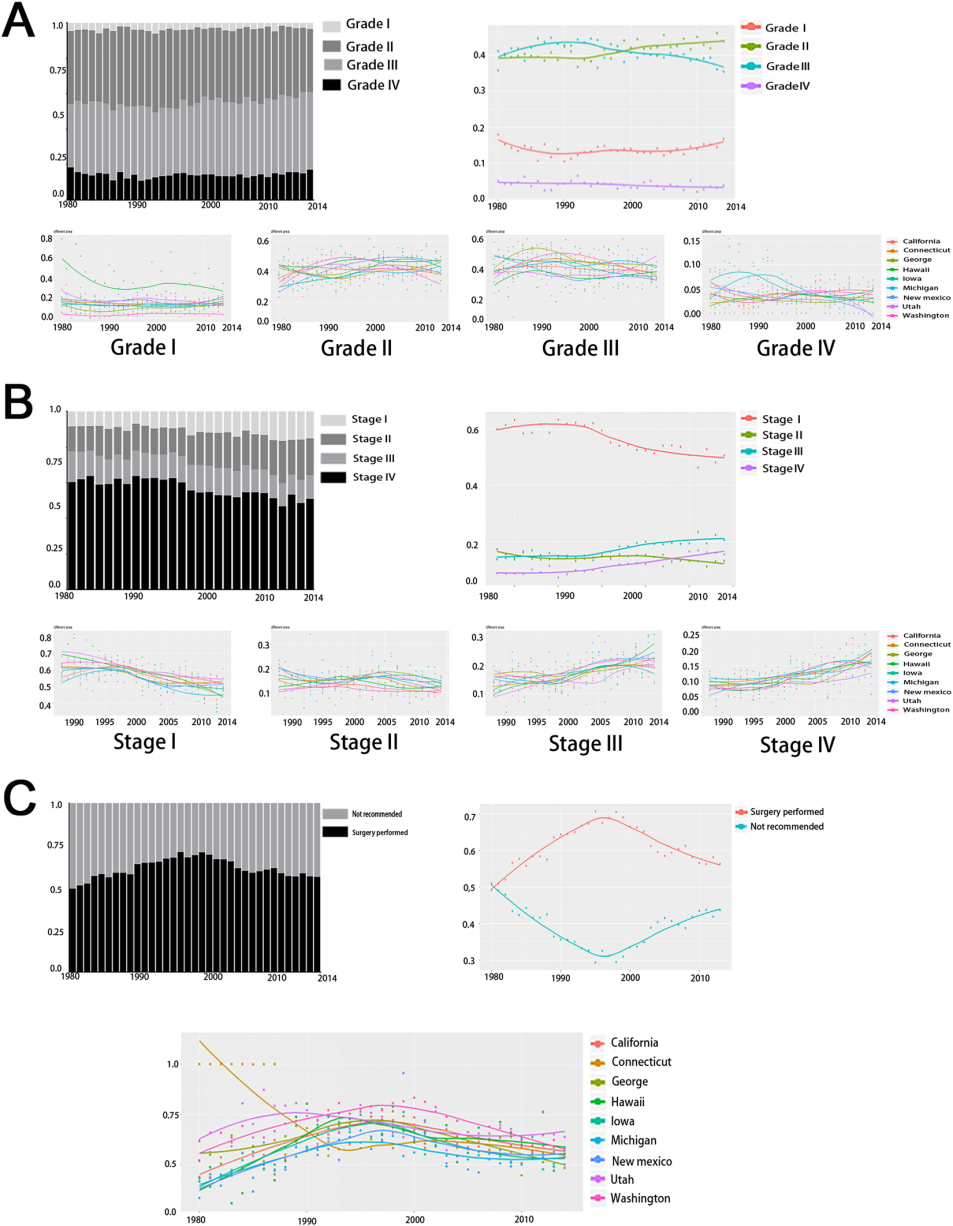
Distribution of cervical cancer cases registered in 9 states according to the mentioned covariates: tumor grade (**A**), tumor stage (**B**) and surgery performed status (**C**) by years during 1980–2014. (**A**) The percent of cervical cancer patients with tumor at each grade by year. Distribution of cervical cases of 9 registered states according to tumor grades from 1980–2014. (**B**) The percent of cervical cancer patients with tumor at each stage by year. Distribution of cervical cases of 9 registered states according to tumor stages from 1980–2014. (**C**) The percent of surgery performed status among cervical cancer cases by year. Distribution of cervical cases of 9 registered states according to surgery performed status from 1980–2014.

Furthermore, we explored decisive factors closely related to patients’ prognosis–diagnosed stages, grades, and surgery performed status counted at a state level in our time series analysis which showed in Fig. 4. According to the variation curves, nearly 80% registered cases were diagnosed at grade II or grade III and more than 60% cases were diagnosed at stage I, which were the majority in the long-term study. For all registered cervical cancer cases, the proportion diagnosed in stage III and stage IV showed up-trend slopes in the long-term. For patients with cervical cancer in different states, patients in Hawaii showed a great downward trend for stage I in the long term and larger proportion were presented for Grade IV in Iowa and Michigan compared with that in other states. Additionally, patients in California, Connecticut, Washington and Utah showed larger proportion in early stages and smaller proportion in later stages. However, more patients in Michigan were diagnosed at later stages compared to other states in the long-term. Meanwhile, patients in California, Connecticut and Washington showed smallest fluctuant range for the variation tendency of tumor diagnosed stages. However, an obvious upward or downward trend was showed for patients in late and early stages respectively in Michigan and New Mexico with years passing by. Primary surgery performed cover rates for the registered cervical cancer cases presented ascension during 1980–1995, and a declining trend in the later years, which was consistent with the variation of 3-year CSS rates for the research population. Obviously, more patients would receive the surgery treatment in Washington, Utah and California in the long term showed in Fig. 4.

## Discussion

Although previous studies showed that the disparities for prevalence, incidence and survival rates of cervical cancer cases existed among the different areas^14,15^. In our long-term time series research, we focused on exploring the potential correlation between the overall area-specific social economic status and local occurrence and development status of cervical cancer using population-based cancer registry data. The differences of incidence rates existed among 9 registered states, which could be attributed to various factors, such as health care expenditure, capital investment of screening programs, exposure to risk factors (e.g. HPV infection) and population coverage of HPV vaccine in different areas^16–19^.

Due to the different status of economic development and health care expenditure in different area, vulnerable population with low income might has less access to HPV vaccine^20^. Furthermore, many African Americans still had lower economic status than other races. In some states such as Connecticut and Michigan where the African Americans were the majority for the cervical cancer cases, showed the slower declining trend or higher incidence rates than other states, perhaps attributing to the lower HPV vaccine coverage for African Americans in these states^21,22^. However, we can also see the great decliningof the incidence rates for African Americans which indicated that local government spared no efforts to improve the healthcare environment for African Americans.

The improvement of screening program was another vital reason for the down-trend slopes of incidence in the long period^23^. During 1980–2014, the GDP and health care expenditure increased a lot, and one of the vital components was hospital service. With the investment of hospital service by U.S. government increasing, more advanced screening methods to prevent cervical cancer replaced the old ones and coverage of the standard screening process among population also expanded a lot in the past years. Therefore, a great declining was presented for the cases from precancerous stages to invasive cervical cancerstages. Previous study showed that, in regard to screening, area-specific socioeconomic status and income were important factors for the success in screening programs promoting^24^. Therefore, the improvement for the screening technology and coverage couldn’t separate from the local economic development and increasing health care expenditure. Our work showed that great disparities of the incidence rates were demonstrated among the different areas. The economic development in some states such as Michigan and New Mexico showed the poorer status compared to other registered states, which perhaps related to their worse outcomes of the cervical cancer. In addition, as the complete treatment strategies healthcare system were conducted many years ago, in recent years, the down-trend slopes showed the slower paces for incidence rates disparities. The patients in the area with better economic condition such as Connecticut, Washington can enjoy more complete Medicare system and advanced screening programs long before, so a fewer fluctuation for incidence rates during 1980–2014, comparing to the states like Michigan and New Mexico.

Previous reports showed most of patients with cervical cancer in early stages (I-II), who accepted proper treatment always gained the great benefits, with only 10% to 15% of the patients suffering recurrence or other bad outcomes^25^. Therefore, the timely and accurate diagnosis to identify the status of cancer development were particularly necessary before treatment. In other words, the earlier patients could be diagnosed, the more benefits would they gained. In our study, the number of patients diagnosed at stage I decreased in these years. One of the important factors was that, with increasing PHC and hospital services expenditure, health department in U.S. draw much attention on preventing risk factors and first screening program^26^. However, the percentages of patients in stage III-IV increased in these years. The deterioration of tumor needs a long period, and the patients firstly diagnosed with stages III-IV must suffered serious adverse reactions on body for a long time. Despite there were great improvements on hospital services construction, through promoting Medicare and Medicaid expenditure, these vulnerable groups were still unable to obtain timely medical diagnosis and treatment, perhaps for their low economic status.

Patients in the states with advanced economic status and more health care expenditure according to GDP index, PHC expenditure, Medicare and Medicaid expenditure, such as in California, Georgia and Utah, had more chances for timely diagnosis. California and Georgia are the top two states for GDP ranking among nine registered states, and the medical strength of California and Utah ranked the top 15 among all the 52 states. In consistence with the mentioned factors, cases with a larger proportion at stage I-II and a less proportion at stage III-IV were presented in these states compared with others. While, compared with these three states, the per capita GDP level in Michigan and New Mexico ranked the 34 and 49 among all the states in U.S., and the health care abilities also located in backward position. Cases in these two states more likely suffered the delayed diagnosis due to the local economic status. Additionally, the rates of cases diagnosed at stage IV in Connecticut and Michigan were the most. In these two states, African Americans were the major components among local registered cases, and these vulnerable people perhaps had lower social or economic position. As a result, they couldn’t get benefits from the advanced medical services. The investment distribution of health care construction by U.S. government should put these people into consideration, so that they make more vulnerable population enjoy the current advanced diagnosis and treatment technology.

We also focus that, patients diagnosed with tumor at grade I in Hawaii showed a great declining than others and rates of cases diagnosed at grade IV also showed the declining slope in our time series analysis, which was closely related to the development of local economic status and healthcare in recent years. Hawaii is different from other traditional rich states such as California and Connecticut. Relying on tourism industry development, Hawaii became a rising star in the later years and medical strength also ranked the first in recent years. Therefore, with the development of economic status, screening programs and timely diagnosis both improved a lot for the local people.

Timely treatment is another vital prognostic factor for the cervical cancer cases^26^. In our research, surgery treatments were divided into radical surgery and conservative surgery, and patients received surgery showed higher 3-year CSS rates (89.8%) than those (54.8%) who were not recommended surgery. After radical trachelectomy(RT) introduced by Dr. Daniel Dragnet in 1987^27^, surgery was the most important factor for outcome of the disease, particularly for cases at early stages^28^. In the current study, despite the social economic status and medical treatment technology improved a lot, the 3-year CSS rates didn’t show the steady up-trend in the long-term. More interestingly, up-trend slopes of 3-year CSS rates were showed from 1980–1995, and in the later years the rates decreased, which showed the same trends for the surgery performed rates among these cervical cancer patients. Although we could see the close relationship between surgery treatments and survival rates, due to various factors, women were less likely to receive surgical treatments in recent years. Contemporarily, more and more women wouldn’t accept the RT treatment, particularly for young women without pregnancy or women had high social status^29^. They would suffer more psychological pressure after RT, so they insisted the conservative treatment or give up receiving any efficient treatments. In addition, the timely diagnosis, Medicare coverage and healthcare expenditure in different states would greatly affect the surgery cover rate. For those less advanced states like Michigan and New Mexico, the proportion of patients received surgery treatment were not satisfactory. Additionally, in these two states, the proportion of cases diagnosed at later stages had a larger increasing trend after 1995 and these cases lost chances for surgery treatment, which could explain the variation trends of surgery cover rates. Compared with Michigan and New Mexico, the patients in California, Hawaii and Washington had better chances for the surgery treatment, so patients there always had better outcomes. Actually, the proportion of patients received the surgery treatments were corresponding to cases’ 3-year CSS rates variation in different states. With postoperative adjuvant therapy^30^, laparoscopic radical surgery and robot-assisted surgery widely applying for the cervical cancer surgery treatment^31–33^, the higher 3-CSS rates for the cases with well or moderate differentiation tumor demonstrated in these economic advanced states. In some areas, African Americans were the majority of the patients who were less likely to receive surgical treatment^34^, and showed worse survival in the long trend.

Because of the higher level of economic condition and more complete healthcare environment construction in California, Connecticut and Washington, patients there would have more  strategies for conservative treatment, comparing with patients in Michigan and New Mexico. Although patients with poorly differentiation tumor or in late stages only took up a small proportion in all registered cases, most of these patients generally lost chances for surgery, so chemoradiotherapy^32,34,35^ became a vital treatment method for them, which draw more attention by the government in U.S. Unfortunately, in many low and middle-income states, radiation therapy capacity is severely limited^36^, leading to the worse prognosis in Michigan and New Mexico.

Our study have some intrinsic limitations. First, we used GDP and per capita GDP to assess local economic development status and made PHC expenditure, Medicare and Medicaid expenditure as indicators for healthcare expenditure geographically. Therefore, some significant indexes could be filtered. Second, individual-level socioeconomic status information was not available in our research, so we cannot link patients’ tumor or treatment information with their individual socioeconomic status. Additionally, all the information of cervical cancer cases was all extracted from SEER database. Therefore, the cohort in the long trend only covered a part of population in U.S. According to the cases’ information provided by registration points among nine states, bias might exist between the computed cases and reality. For example, in some states, only several cases were registered in specific period. Due to the small numbers of race-combined with region-specific analysis, the latter finding should require future validation.

In summary, our research showed that the differences in variation tendency of prevalence, incidence and outcome status existed among different areas in our time series analysis. The variation were greatly affected by area-specific economic development and healthcare expenditure investment, which connected with timely diagnosis and receiving proper treatment for the local patients. In order to make more patients gain benefits from the treatment, the government in U.S. should break the unbalance status and improve the coverage for the screening, timely accurate diagnosis and proper treatment in low or middle-income areas.

## Methods

### Data resources

A total of 39160 cases diagnosed with cervical cancer were extracted from the National Cancer Institute Survival, Epidemiology, and End Results (SEER) database, a population-based cancer registration program for the years 1980–2014. The registries in our research currently covered approximately 28% of the U.S. population and also had a wide geographic coverage. Nine SEER registry states (California, Connecticut, Georgia, Hawaii, Iowa, Michigan, New Mexico, Utah and Washington) were included in our long trend disease-relevant study. Cervical cancer was identified though histology diagnosis using the International Classification of Diseases (ICD)-10 codes. Local overall economic condition were estimated by GDP index, PHC expenditure, Medicare expenditure and Medicaid expenditure were used to assess area-specific healthcare expenditure, which were all sourced from CSM.gov from1980 to 2014 annually. Additionally, GDP status in different area sourced from U.S. Bureau of Labor Statistics https://www.bls.gov/, and the local medical strength ranking were obtained from www.businessinsider.com. Patients younger than twenty years old were excluded.

### Covariates

Cervical cancer relevant information and patients’ demographic data, which might influence incidence and outcome of the disease, were included in our time series analysis. The prevalence rates were the frequency of patients diagnosed with cervical cancer within the specified period of time among all the people enrolled in our survey, including newly discovered and previous cases. The incidence rates were the frequency of patients just newly diagnosed with cervical cancer within the prescribed period of time among all the registered population. 3-year cervical cancer CSS rates represented survival status of the specific cause of primary cervical cancer death in the absence of other causes of death. The collected patients were all diagnosed during 1980–2014 and they were stratified by 5-year intervals (1980–1984, 1985–1989, 1990–1994, 1995–1999, 2000–2004, 2005–2009, 2010–2014), ages at diagnosis (20–39, 40–59, 60+), race/ethnicity (Caucasians, African Americans, others), registered states (California, Connecticut, Georgia, Hawaii, Iowa, Michigan, New Mexico, Utah and Washington), and surgery performed condition. Information of the tumor included histological diagnosis according to ICD-03 (squamous cell carcinoma, adenocarcinoma and other types), patient’s American Joint Committee on Cancer (AJCC) stages manual (level I–IV), and tumor grades (well-I, moderately-II, poorly-III and undifferentiated-IV). The area-specific economic status and health care expenditure condition were calculated by using GDP, local PHC expenditure, Medicare expenditure and Medicaid expenditure separately for their corresponding registry states.

### Statistical analysis

To evaluate the disparities of happening and outcome of cervical cancer in long term, the prevalence, incidence and 3-year CSS rates were calculated after adjustment for ages and calculated cases per 100,000 women to the 2000 US Standard Population by using SEER-Stat software. Annual percent changes (APCs)^37^ and their 95% CIs were calculated to characterize the variation level by fitting Join point regression which was built into the SEER-Stat software. We regarded the different time period as the unidirectional ordered variables in our time series analysis. For some indicators related to prevalence and incidence, Wilcoxon rank-sum test were utilized to identify the differences in the composition ratio among the disorder classification variable groups, and the Linear-by-linear Association test were applied to examine the differences in the composition ratio among the ordered variable groups over time. For indicators related to survival, we used the Friedman test to do relevant statistical computation. Pearson- correlation analysis was used to assess the association between incidence rates, 3-year CSS rates and area-specific economic factors, including GDP, PHC expenditure, Medicare expenditure and Medicaid expenditure. Locally weighted scatterplots smoothing (LOESS) is a widely used method for smoothing two-dimensional scatterplots, which was suitable for depicting the variation of incidence or 3-year CSS rates in our time series analysis. The geographic heat map was colored according to the GDP rank list in the recent years from “www.countryeconomy.com”. Both the smoothing curves and geographic heat map were conducted by R software (version 3.5.0, 2018). Resting all statistical analysis were performed by using Graph Pad Prism 6.0. We defined statistical significance as alpha (*α*) < 0.05 based on the two-sided significance level.

## Supplementary information

Supplementary Figures

Supplementary Tables
