# Supplementary material for: Area-specific economic status should be regarded as a vital factor affecting the occurrence, development and outcome of cervical cancer
Source: Sci Rep. 2020 Mar 16;10:4759. doi: 10.1038/s41598-020-61660-5 (PMC7075972; doi:10.1038/s41598-020-61660-5)
Supplement: Supplementary file 1 — Supplementary Figures [file 41598_2020_61660_MOESM1_ESM.pdf]

Area-specific economic status should be regarded as a vital factor affecting the occurrence, development and outcome of cervical cancer

Zichao Li[a,b,1], Haozhi Wu[a,d,1], Xiaowei Yi[c,1], Fangyu Tian[e], Xiyang Zhang[a], Haikun Zhou[a,d], Biqing Liu[a] , Zhenhua Lu[a], Jing Wang[a], Dongbo Jiang[a], Lei Shang[f,**], Kun Yang[a,*]

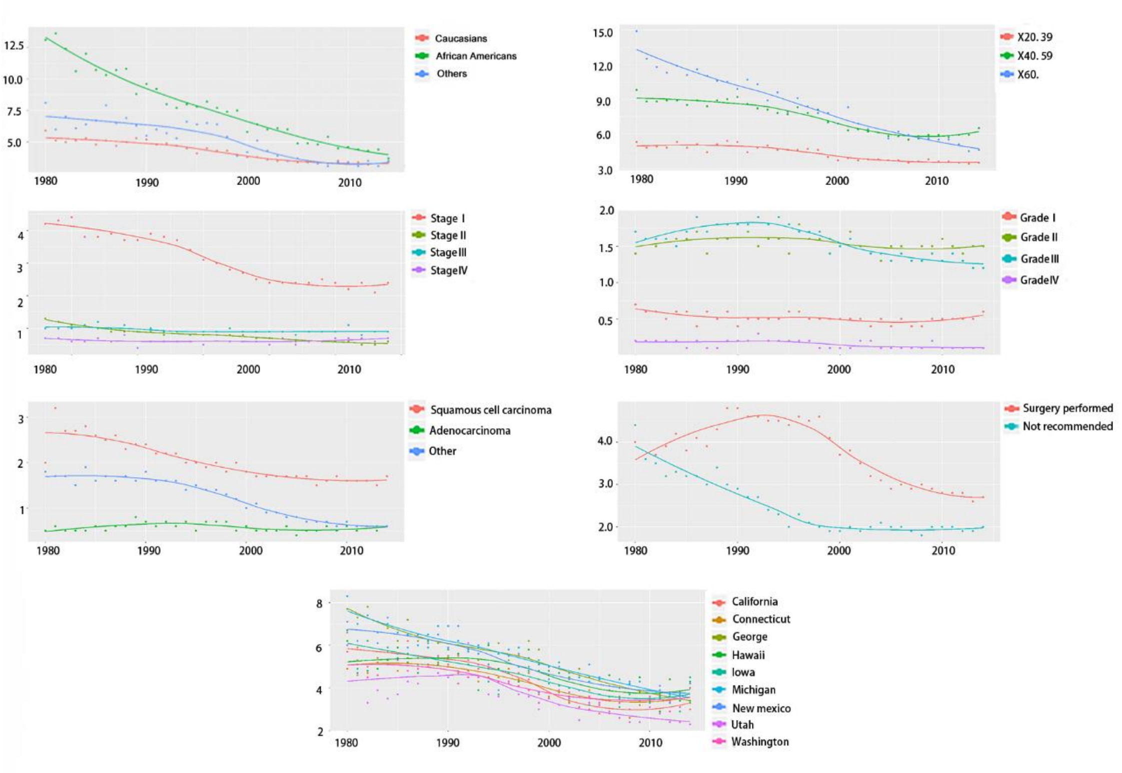

SupplementaryFigure 1.**The variation tendency of age-adjusted incidence rates of registered patients with cervical cancer during 1980-2014 according to following variables: race/ethnicity, ages at diagnosis, tumor staging (AJCC), tumor differentiation status, tumor histology, surgery performed status and registry states.**

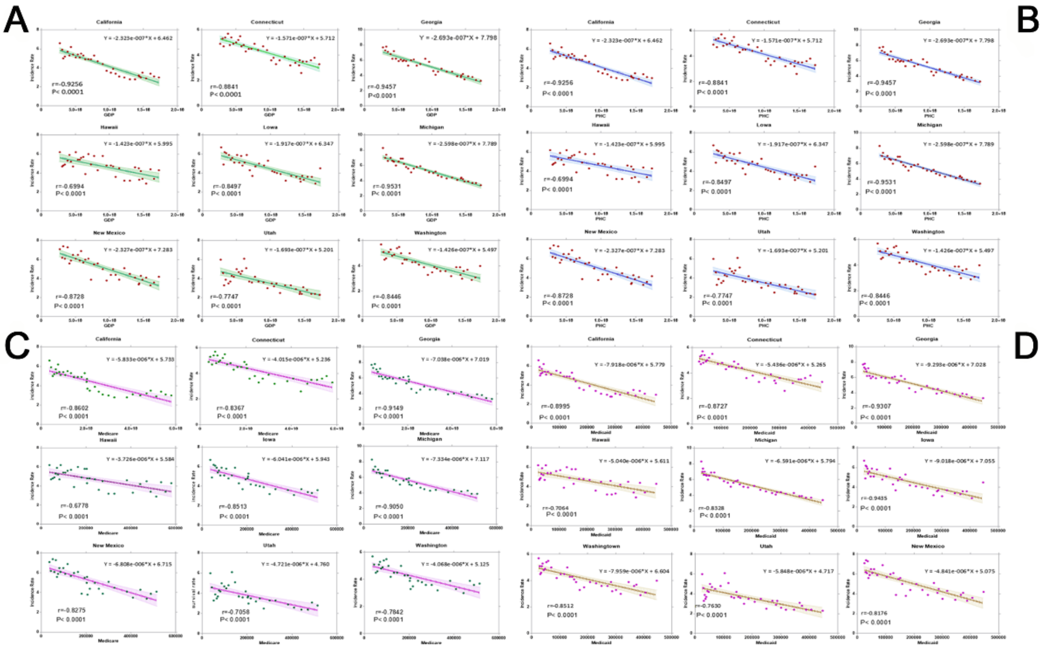

SupplementaryFigure 2.**The correlation between the variation of age-adjusted incidence rates for patients with cervical cancer and four social economic index: local GDP status (A), PHC expenditure (B), Medicare expenditure(C) and Medicaid expenditure (D) were depicted among 9 states between 1980 and 2014.**

a. The correlation was calculated by Pearson correlation.

b. Relationship among variables was evaluated by linear regression, and the shadow showed 95%confidence intervalsfor incidence disparities.

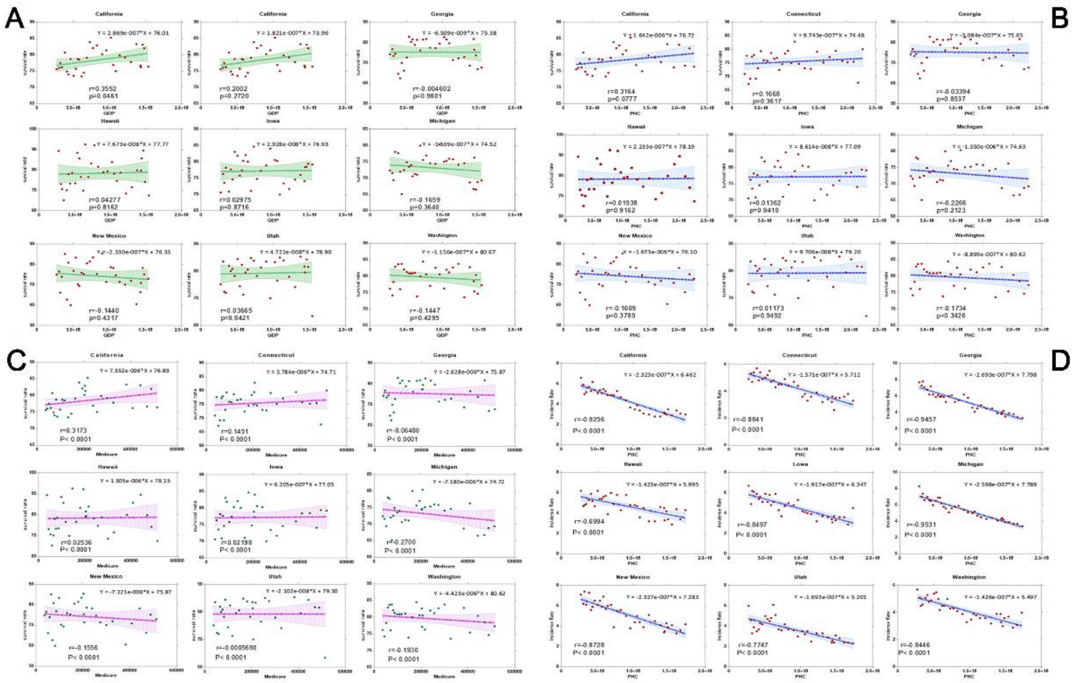

Supplementary Figure 3.**The correlation between the variation of 3-year cause specific survival rates for patients with cervical cancer and four social economic index: local GDP**

status(A), PHC expenditure(B), Medicare expenditure(C) and Medicaid expenditure(D) were depicted among 9 states between 1980 and 2014.

a. The correlation was calculated by Pearson correlation.

b. Relationship among variables was evaluated by linear regression, and the shadow showed 95% confidence intervals for 3-year CSS rates disparities.
