# Supplementary material for: Area-specific economic status should be regarded as a vital factor affecting the occurrence, development and outcome of cervical cancer
Source: Sci Rep. 2020 Mar 16;10:4759. doi: 10.1038/s41598-020-61660-5 (PMC7075972; doi:10.1038/s41598-020-61660-5)
Supplement: Supplementary file 2 — Supplementary Tables [file 41598_2020_61660_MOESM2_ESM.pdf]

Area-specific economic status should be regarded as a vital factor affecting the occurrence, development and outcome of cervical cancer

Zichao Li[a,b,1], Haozhi Wu[a,d,1], Xiaowei Yi[c,1], Fangyu Tian[e], Xiyang Zhang[a], Haikun Zhou[a,d], Biqing Liu[a] , Zhenhua Lu[a], Jing Wang[a], Dongbo Jiang[a], Lei Shang[f,**], Kun Yang[a,*]

Supplementary Table 1. Age-standardized cervical cancer prevalence rates among women ages 20+ in 9 states during 1980-2014

		1980-1984		1985-1989		1990-1994		1995-1999		2000-2004		2005-2009		2010-2014		P
		Count	Rate（%）	Count	Rate（%）	Count	Rate（%）	Count	Rate（%）	Count	Rate（%）	Count	Rate（%）	Count	Rate（%）	Rate（%）
**Race**	Caucasians	3,272	0.01948	3,352	0.01844	3,688	0.01871	3,504	0.01683	3,075	0.01448	2,769	0.01282	2,784	0.01260	<0.001
	African	638	0.04024	621	0.03451	601	0.02781	647	0.02602	590	0.02129	533	0.01666	482	0.01374	
	Others	327	0.02521	381	0.02353	419	0.02061	569	0.02252	480	0.01565	434	0.01202	486	0.01158	
**Ages**	20-39	1,453	0.00648	1,633	0.00642	1,763	0.00637	1,690	0.00602	1,398	0.00522	1,209	0.00466	1,191	0.00450	< 0.001
	40-59	1,605	0.00911	1,578	0.00838	1,833	0.00844	2,064	0.00804	1,921	0.00660	1,773	0.00567	1,823	0.00577	
	60+	1,192	0.00598	1,157	0.00530	1,140	0.00484	1,017	0.00419	851	0.00333	782	0.00275	796	0.00234	
**Histology**	Squamous cell	2,020	0.01032	1,842	0.00853	1,835	0.00772	1,862	0.00716	1,788	0.00651	1,711	0.00590	1,753	0.00576	<0.001
	Adenoma	348	0.00182	461	0.00220	603	0.00253	665	0.00255	578	0.00209	582	0.00206	660	0.00223	
	Others	1,309	0.00652	1,497	0.00678	1,607	0.00656	1,482	0.00565	1,127	0.00411	788	0.00275	705	0.00233	
**Stages**	Stages I					3,138	0.01284	3,228	0.01224	2703	0.00811	2,218	0.00785	2,213	0.00757	< 0.001
	Stages II					569	0.00247	566	0.00223	620	0.00180	503	0.00169	464	0.00145	

	Stages III					506	0.00215	504	0.00195	618	0.00166	593	0.00203	673	0.00216	
	Stages IV					146	0.00063	170	0.00066	248	0.00063	174	0.00057	258	0.00079	
**Grade**	Grade I	317	0.00164	303	0.00143	340	0.00145	449	0.00171	423	0.00154	391	0.00138	489	0.00166	<0.001
	Grade II	760	0.00390	865	0.00404	1,018	0.00425	1,137	0.00436	1,205	0.00436	1,131	0.00393	1,290	0.00428	
	Grade III	702	0.00364	812	0.00381	989	0.00418	1,098	0.00424	951	0.00345	944	0.00323	917	0.00294	
	Grade IV	62	0.00031	52	0.00024	89	0.00038	79	0.00030	82	0.00030	66	0.00022	72	0.00023	
**State**	California	678	0.02120	711	0.02012	758	0.01969	777	0.01847	605	0.01383	531	0.01160	592	0.01209	<0.001
	Connecticut	555	0.01802	612	0.01885	604	0.01794	616	0.01751	522	0.01412	461	0.01247	490	0.01328	
	Georgia	402	0.02607	370	0.02008	467	0.02090	548	0.02151	501	0.01779	445	0.01407	447	0.01258	
	Hawaii	170	0.01979	187	0.01908	231	0.02005	249	0.02051	214	0.01668	194	0.01419	201	0.01456	
	Iowa	598	0.02287	557	0.02115	584	0.02090	490	0.01703	476	0.01636	399	0.01336	387	0.01271	
	Michigan	904	0.02619	841	0.02293	853	0.02187	827	0.02044	717	0.01753	609	0.01538	532	0.01344	
	New Mexico	286	0.02473	302	0.02285	330	0.02159	341	0.01983	294	0.01602	289	0.01462	280	0.01360	
	Utah	183	0.01612	213	0.01611	283	0.01834	259	0.01467	230	0.01158	234	0.01040	243	0.00966	
	Washington	487	0.01858	596	0.01917	639	0.01752	678	0.01667	621	0.01439	615	0.01331	645	0.01289	

Source: SEER-NLMS Record Linkage Study. Based on the registered population among 9 SEER Registries (California, Connecticut, Georgia, Hawaii, Iowa, Michigan, New Mexico, Utah and Washington) during 1980-2014.

a.Rates were counted per 100,000 population and age-adjusted to the 2000 US standard population by the direct method.

b.Rates were estimated by Join point regression models which was adjusted by ages.

c.Wilcoxon rank-sum tests were used to identify the different variation of prevalence among different races, age groups, histology types, and states over time; Linear-by-Linear association tests were used to identify the different variation of prevalence among tumor differentiated stages and grades over time.

Supplementary Table 2. The annual percent changes (APC) rates for cervical cancer incidence rates among registered patients during 1980-2014

		Trend1		Trend2		Trend3		Trend4		1980-2014
		YEARS	APC	YEARS	APC	YEARS	APC	YEARS	APC	AAPC
**ALL**		1980-1987	-1.9*	1988-1995	-2.7*	1996-2003	-4.2*	2004-2014	-0.7*	-2.0*
**Race**	Caucasians	1980-1987	-1.9*	1988-1995	-2.8*	1996-2003	-3.7*	2004-2014	-0.5*	-1.7*
	African Americans	1980-1987	-3.6*	1988-1995	-4.2*	1996-2003	-4.7*	2004-2014	-3.3*	-3.5*
	Others	1980-1987	-0.1*	1988-1995	-0.1*	1996-2003	-6.6*	2004-2014	-0.4*	-2.8*
**Ages**	20-39	1980-1987	-1.0*	1988-1995	-2.0*	1996-2003	-3.6*	2004-2014	-0.8*	-1.3*
	40-59	1980-1987	-1.3*	1988-1995	-2.6*	1996-2003	-4.6*	2004-2014	0.6*	-1.7*
	60+	1980-1987	-3.2*	1988-1995	-3.2*	1996-2003	-3.9*	2004-2014	2.7*	-3.0*
**Histology**	Squamous cell carcinoma	1980-1987	-3.0*	1988-1995	-3.3*	1996-2003	-2.6*	2004-2014	-0.3*	-1.9*
	Adenocarcinoma	1980-1987	-2.5*	1988-1995	-3.6*	1996-2003	-4.9*	2004-2014	-3.9*	-0.3*
	Others	1980-1987	-0.9*	1988-1995	-2.7*	1996-2003	-6.7*	2004-2014	-2.9*	-3.6*
**Surgery**	Surgery performed	1980-1987	0.8	1988-1995	-0.6	1996-2003	-5.1	2004-2014	-1.2	-1.5
	Not recomended	1980-1987	-4.3	1988-1995	-6.6	1996-2003	-1.8	2004-2014	-0.1	-2.2
**Stages**	Stages I	1980-1987		1988-1995	-2.5	1996-2003	-5.4	2004-2014	-0.8	

	Stages II	1980-1987		1988-1995	-5.7	1996-2003	-2.4	2004-2014	-2.9	
	Stages III	1980-1987		1988-1995	-0.8	1996-2003	-0.2	2004-2014	0.5	
	Stages IV	1980-1987		1988-1995	-1.5	1996-2003	1.8	2004-2014	2.6	
**Grade**	Grade Ⅰ	1980-1987	-2.6	1988-1995	2.0	1996-2003	-0.4	2004-2014	2.5	-0.4
	Grade Ⅱ	1980-1987	0.7	1988-1995	-1.6	1996-2003	-1.8	2004-2014	0.6	-0.2
	GradeⅢ	1980-1987	1.4	1988-1995	0.2	1996-2003	-3.0	2004-2014	-1.5	-1.1
	GradeⅣ	1980-1987	-1.9	1988-1995	3.2	1996-2003	-0.1	2004-2014	-0.9	-1.9
**State**	California	1980-1987	-0.9*	1988-1995	-1.1*	1996-2003	-6.7*	2004-2014	0.9*	-2.3*
	Connecticut	1980-1987	0.3*	1980-1987	-2.6*	1996-2003	-5.4*	2004-2014	0.1*	-1.6*
	Georgia	1980-1987	-2.7*	1988-1995	-0.2*	1996-2003	-3.4*	2004-2014	-2.6*	-2.3*
	Michigan	1980-1987	-3.5*	1988-1995	-3.8*	1996-2003	-3.5*	2004-2014	-2.1*	-2.2*
	Iowa	1980-1987	-2.3*	1988-1995	-4.2*	1996-2003	-3.5*	2004-2014	1.0*	-1.9*
	New mexico	1980-1987	-1.7*	1988-1995	-3.6*	1996-2003	-4.0*	2004-2014	-2.1*	-2.0*
	Washington	1980-1987	-1.1*	1988-1995	-2.7*	1996-2003	-1.8*	2004-2014	0.0*	-1.5*
	Utah	1980-1987	-3.3*	1988-1995	-3.0*	1996-2003	-4.5*	2004-2014	-3.0*	-2.2*
	Hawaii	1980-1987	-1.1*	1988-1995	-2.0*	1996-2003	-3.9*	2004-2014	1.0	-1.4*

Source: SEER-NLMS Record Linkage Study. Based on the registered population among 9 SEER Stares (California, Connecticut, Georgia, Hawaii, Iowa, Michigan, New Mexico, Utah and Washington) during 1980-2014

a.APC were counted per 100,000 population and age-adjusted to the 2000 US standard population by the direct method.

b.APC were estimated by Join point regression models which was adjusted by ages.

Supplementary Table 3. Age-standardized cervical cancer 3-year survival rates and 95% confidence intervals (CI) among women ages 20+ in 9 states during 1980-2014

		1980-1984			1985-1989			1990-1994			1995-1999			2000—2004			2005—2009			2010-2014			1980-2014			*P*
		rate	CI		rate	CI		rate	CI		rate	CI		rate	CI		rate	CI		rate	CI		rate	CI		
			low	up		low	up		low	up		low	up		low	up		low	up		low	up		low	up	
All		75.0	73.8	76.2	75.6	74.4	76.7	74.4	73.2	75.9	79.6	78.5	78.5	77.4	76.1	78.5	76.1	74.8	77.4	75.0	73.4	76.5	76.6	76.2	77.1	
Race	Caucasians	77.5	77.0	78.0	75.3	73.9	76.6	77.2	75.9	78.5	78.1	76.8	79.3	80.0	78.7	81.3	77.7	76.3	79.1	77.2	75.6	78.6	77.5	77.0	78.0	
	African Americans	71.3	68.1	74.2	65.2	61.7	68.4	69.0	65.5	72.2	73.7	70.5	76.6	73.8	70.3	76.9	67.8	66.7	71.3	64.3	59.4	68.7	69.6	68.2	70.8	＜0.001
	Other	83.0	83.0	86.5	82.2	78.4	85.4	82.0	82.9	79.5	85.7	79.1	75.4	82.3	78.7	85.3	82.2	80.9	83.5	76.3	72.1	80.0	82.2	80.9	83.5	
Ages	20-39	89.8	86.0	92.6	85.5	81.6	89.5	86.2	84.4	87.8	88.0	86.2	89.5	88.6	86.6	90.3	86.5	84.5	88.3	87.3	84.6	89.3	89.8	89.0	90.4	
	40-59	75.4	73.4	77.3	76.1	74.1	77.9	78.2	76.4	79.9	79.8	78.1	81.4	77.0	75.2	78.8	76.9	75.0	78.7	76.6	74.3	78.7	75.0	74.2	76.6	＜0.001
	60+	62.6	60.2	64.9	62.6	60.3	65.1	63.2	60.7	65.7	67.0	64.3	69.6	63.1	60.2	65.9	61.1	58.0	64.0	58.3	54.6	61.8	62.2	61.9	63.9	

Histology	Squamous cell carcinoma	72.4	70.6	74.1	70.9	69.0	72.0	73.3	71.4	75.1	77.3	75.5	79.0	75.4	73.4	77.3	74.2	72.2	76.1	72.7	70.3	75.0	73.7	73.0	74.5	< 0.001
	Adenocarcinoma	80.2	78.9	81.4	74.6	70.1	78.4	80.0	78.6	83.0	77.8	74.5	80.6	84.5	81.5	87.0	79.6	76.2	82.5	80.5	76.7	83.7	82.4	79.3	85.2	
	Others	79.5	77.5	81.4	81.5	79.5	83.3	82.5	80.6	84.2	82.7	80.7	84.5	75.5	70.7	80.6	78.2	74.8	82.8	80.4	76.8	83.5	81.1	80.2	81.9	
Surgery	performed	89.6	88.3	90.7	89.4	88.2	90.4	88.6	87.6	89.6	90.1	89.1	91.0	90.5	89.4	91.5	90.3	89.1	91.4	90.5	89.0	91.8	89.8	89.4	90.2	< 0.001
	Not recommended	60.1	58.0	62.2	56.0	53.8	58.2	52.7	50.3	55.0	54.0	51.4	56.5	52.0	49.4	54.4	53.9	51.5	56.3	53.3	50.5	56.1	54.9	54.0	55.8	
Stages	Stages I				94.1	92.6	95.2	93.8	92.9	94.6	94.6	93.8	95.4	94.3	93.0	95.3	94.2	93.1	95.1	95.1	93.8	96.2	94.2	93.7	94.7	< 0.001
	Stages II				68.4	63.1	73.2	69.6	66.0	72.9	71.9	68.3	75.1	76.0	71.1	80.1	74.5	70.6	77.9	79.9	75.3	83.8	71.5	69.6	73.4	
	Stages III				52.9	46.9	58.6	52.6	49.0	56.1	55.6	51.9	59.2	61.7	56.9	66.2	64.8	61.3	68.1	64.5	60.5	68.3	55.9	53.9	57.9	
	Stages IV				16.5	11.3	22.5	20.7	17.1	24.6	24.1	20.1	28.3	23.1	18.5	28.2	22.5	18.8	26.3	26.7	22.5	31.0	22.2	20.1	24.4	
Grade	Grade I	80.9	76.4	84.6	84.2	79.9	87.6	83.1	78.9	86.6	91.9	88.9	94.0	91.4	88.2	93.7	91.0	87.9	93.4	95.3	92.4	97.1	88.5	87.2	89.6	< 0.001
	Grade II	73.0	70.1	75.7	72.5	69.7	75.1	77.1	74.6	79.4	78.6	76.2	80.7	78.8	76.5	80.9	78.8	76.5	81.0	81.0	78.3	83.3	77.3	76.4	78.2	
	Grade III	62.9	59.7	65.8	62.0	59.2	64.7	63.3	60.7	65.8	67.5	64.9	70.0	66.7	63.9	69.3	66.1	63.3	68.7	63.0	59.6	66.2	64.6	63.5	65.6	
	Grade IV	47.6	38.0	56.6	43.4	33.1	53.2	57.4	48.6	65.1	55.3	46.0	63.6	55.7	46.2	64.2	62.7	52.5	71.3	63.5	51.8	73.2	54.6	50.9	58.1	
State	California	77.3	74.1	80.1	75.4	72.3	76.2	76.9	73.9	79.6	82.1	79.3	84.6	79.4	75.8	82.1	80.3	76.8	83.3	80.3	76.5	83.5	78.5	77.3	79.6	<

Connecticut	72.8	69.3	75.9	75.1	71.7	78.1	76.0	72.6	79.1	78.1	74.8	81.0	75.0	71.3	78.4	76.7	72.7	80.2	75.0	70.2	79.1	75.5	74.2	76.8	0.001
Georgia	72.8	68.6	76.5	71.3	67.1	75.0	79.6	76.0	82.8	79.5	76.1	82.4	84.1	73.6	80.6	74.8	70.8	78.3	68.6	63.1	73.5	75.5	74.0	76.8	
Hawaii	74.5	67.7	80.1	78.4	72.7	83.1	79.7	74.3	84.1	83.3	78.3	87.2	77.2	71.2	82.1	81.7	76.1	86.2	75.3	68.2	81.0	78.8	76.8	80.8	
Iowa	73.7	73.7	76.8	78.6	75.3	81.6	78.4	75.0	81.3	79.9	76.4	82.9	75.4	71.3	78.9	76.3	72.2	79.9	75.5	70.6	79.8	76.8	75.5	78.1	
Michigan	73.8	71.1	76.3	71.7	68.8	74.3	73.3	70.4	76.0	76.4	73.5	78.9	75.3	75.3	78.2	70.6	67.1	73.8	70.5	66.0	74.5	73.3	62.4	65.9	
New Mexico	75.9	70.9	80.1	73.4	68.4	77.7	74.4	69.9	78.3	79.8	75.2	83.6	74.8	70.0	78.9	69.8	64.5	74.4	74.0	67.7	79.2	74.7	72.9	76.4	
Utah	77.2	71.2	82.2	79.8	74.3	84.2	79.6	74.9	83.5	79.6	74.5	83.7	81.6	76.4	85.7	80.9	75.6	85.1	77.1	70.5	82.4	79.5	77.6	81.3	
Washington	79.1	75.6	82.1	80.8	77.7	83.5	79.4	76.2	82.2	81.1	77.9	83.8	81.1	77.9	83.8	81.2	75.1	81.2	77.6	73.8	81.0	79.8	78.6	80.9	

Source: SEER-NLMS Record Linkage Study. Based on the registered population among9 SEER Stares (California, Connecticut, Georgia, Hawaii, Iowa, Michigan, New Mexico, Utah and Washington) during 1980-2014

a.Rates were age-adjusted to the 2000 US standard population by the direct method.

b.Rates were estimated by Join point regression models which was adjusted by ages.

c.Friedman test were used to make statistical test.

d.Confidence interval level was 95%.
